# *CYP2C9* Promoter Variable Number Tandem Repeat Polymorphism in a Dominican Population: Exploring Differences with Genetic Ancestry

**DOI:** 10.3390/genes16050540

**Published:** 2025-04-30

**Authors:** Carla González de la Cruz, Mariela Guevara, Fernanda Rodrigues-Soares, Ernesto Rodríguez, Eva Peñas-Lledó, Adrián LLerena, Pedro Dorado

**Affiliations:** 1 Personalized Medicine and Mental Health Unit, INUBE University Institute for Bio-Sanitary Research of Extremadura, 06080 Badajoz, Spain; carla.gonzalezd@externos.salud-juntaex.es (C.G.d.l.C.); fernanda.soares@uftm.edu.br (F.R.-S.); elledo@unex.es (E.P.-L.); allerena@unex.es (A.L.); 2 Pharmacogenetic and Pharmacogenomics Unit, Extremadura Health Service, Badajoz University Hospital, Extremadura University, 06080 Badajoz, Spain; 3 Research and Development Department, Universidad Nacional Pedro Henríquez Ureña, Santo Domingo 10203, Dominican Republic; ernest2288@yahoo.es (M.G.); er18-2087@unphu.edu.do (E.R.); 4 Department of Pathology, Genetic and Evolution, Biological and Natural Sciences Institute, Universidade Federal do Triângulo Mineiro, Uberaba 38025-350, Brazil; 5 Department of Therapeutics and Medical-Surgery, Medical and Health Sciences School, 06006 Badajoz, Spain

**Keywords:** *CYP2C9*, pVNTR, linkage disequilibrium, ancestry

## Abstract

A variable number tandem repeat polymorphism has been described in the *CYP2C9* promoter (pVNTR) with three types of fragments: short (*pVNTR-S*), medium (*pVNTR-M*), and long (*pVNTR-L*). The *pVNTR-S* allele appears in strong linkage disequilibrium (LD) with the non-functional CYP2C9*3 allele in populations of European ancestry, but independently of this, it also appears to reduce the level of CYP2C9 expression in human liver by up to 34%. Objectives: This study, in a Dominican population with varying amounts of Western European, African, and Native American ancestry, aims primarily to determine the frequency of *CYP2C9* pVNTR, and the degree of LD of *pVNTR-S* with CYP2C9*3. Secondarily, it explores if the frequency of the *pVNTR-S* allele is over- or under-represented in those with a greater component of African ancestry. Methods: A total of 193 healthy volunteers from the Dominican Republic participated in the study. The promoter region of *CYP2C9* was amplified and analyzed by capillary electrophoresis. Analyses of *CYP2C9* genotypes (*2, *3, *5, *6, and *8) and genetic ancestry, estimated in 176 Dominican individuals by genotyping 90 ancestry informative markers, were previously performed in this population. Results: The frequencies of *CYP2C9 pVNTR-L*, *M,* and *S* variants are 0.065, 0.896, and 0.039, respectively. LD between *pVNTR-S* and CYP2C9*3 was found (D’ = 0.756, r^2^ = 0.702) to be weaker than in European populations. Conclusions: Populations with a greater African ancestry component appear to present a lower-than-expected frequency of *pVNTR-S*, as well as a lower tendency for this and CYP2C9*3 alleles to be inherited together, as is common in Europeans. The present exploratory results warrant further research in vivo about the effects of *pVNTR-S* in predicting *CYP2C9* activity. Its inclusion in *CYP2C9* testing panels for personalized drug therapy could be relevant in populations such as the Dominican, where the LD between *pVNTR-S* and CYP2C9*3 is low.

## 1. Introduction

The enzyme responsible for the metabolism of about 20% of drugs is CYP2C9, which belongs to the P450 enzyme complex. It is involved in the metabolism of drugs widely used in today’s clinical practice, such as non-steroidal anti-inflammatory drugs (ibuprofen, celecoxib, and diclofenac) [1], anticoagulants (acenocoumarol and warfarin) [2], antiepileptics (phenytoin) [3], and statins (fluvastatin) [4]. The *CYP2C9* gene is located at 10q23.33 within the locus where other genes such as *CYP2C19*, *CYP2C18*, and *CYP2C8* are located [5].

This enzyme is composed of 490 amino acids with a size of 55 kDa, and its level of expression is mainly found in the liver [6]. The *CYP2C9* gene is highly polymorphic, with a total of 85 variants currently described [7], although there are variants with greater relevance that have been widely studied, such as CYP2C9*2 (rs1799853) and CYP2C9*3 (rs1057910), associated with a decrease or null enzyme activity, respectively [8].

This partial or total loss of enzymatic function has been demonstrated in different studies of plasma levels with drugs such as diclofenac, losartan, phenytoin, tolbutamide, or warfarin, with higher plasma concentrations in those individuals with CYP2C9*2 and/or CYP2C9*3 compared to CYP2C9*1/*1 individuals [9,10,11,12]. However, interethnic variability in CYP2C9 metabolism remains unexplained, mainly in individuals of non-European ancestry.

A variable number tandem repeat polymorphism (pVNTR) is present in the promoter region of the *CYP2C9* gene, specifically, 4 Kb upstream of the translation start site. There are three pVNTRs with different fragment lengths: long (*pVNTR-L*), medium (*pVNTR-M*), and short (*pVNTR-S*).

This promoter polymorphism affects CYP2C9 mRNA expression in the liver [13], specifically, *pVNTR-S* is associated with lower CYP2C9 mRNA expression, reducing the transcriptional activity of the gene compared to *pVNTR-M* and *pVNTR-L* [13]. As the total mRNA level is strongly influenced by trans-active factors that may confound the effect of cis-acting polymorphisms, a previous study [13] measured the relative amount of mRNA derived from each of the two alleles in the same individual, finding evidence about the presence of one or more cis-acting regulatory polymorphisms affecting the *CYP2C9* mRNA level by influencing transcription or RNA processing.

It has been reported that the *pVNTR-S* allele is associated with a 25–60% reduction in CYP2C9 mRNA expression compared with the *pVNTR-M* or *pVNTR-L* alleles, respectively [13], and recently, *pVNTR-S* has been associated with lower CYP2C9 expression (34% reduction) in human liver samples [14]. This evidence suggests that *pVNTR-S* reduces CYP2C9 expression, regardless of the presence or not of other CYP2C9 decreased function variants such as CYP2C9*3.

In addition, previous studies in the European population have demonstrated the existence of a near perfect linkage disequilibrium of *pVNTR-S* with CYP2C9*3 [13,14,15], but it has not been observed in African American, Egyptian [13], nor Jordanian populations [16]. This may be due to the ancestral component of each population and the importance of taking it into account when performing genetic analysis [17].

On the other hand, so far, the presence of this polymorphism has not been studied in Latin Americans, whose populations present an admixture, to a greater or lesser extent, of American, African, and European ancestry components.

Therefore, the main objective of this study was to evaluate the presence and frequency of *CYP2C9* pVNTR polymorphisms in the Dominican population, the degree of LD between the *pVNTR-S* and CYP2C9*3 alleles, and to preliminarily explore its potential relationship across different components of genetic ancestry.

## 2. Materials and Methods

### 2.1. Subjects

This study involved 193 unrelated students and employees of the Dominican Republic’s Universidad Nacional Pedro Henríquez Ureña (UNPHU) in Santo Domingo (Dominican Republic). These subjects were participants in a previous study [18]. None of the participants were immigrants, which applied to at least two previous generations; their ages ranged from 20 to 48 years, and 64.2% were women.

The study complied with the principles of the Declaration of Helsinki for human research and was approved by the Ethics Committee of the National Health Bioethics Council (Ref. 018-2022) of the Dominican Republic. All patients’ informed consents were collected in writing at the time of sample collection.

### 2.2. CYP2C9 Genotyping and Genetic Ancestry Analysis

The analyses of the *CYP2C9* genotype in the present Dominican population were previously performed in a study of our group [18]. Genotyping for the *CYP2C9* variants (*2, *3, *5, *6, *8) was conducted using a fluorescence-based allele-specific TaqMan allelic discrimination assay (Thermo Fisher Scientific, Waltham, MA, USA). PCR amplification for all single-nucleotide polymorphisms was conducted in 20 µL reactions containing 30 ng of template DNA, 1× if each primer and probe assay, 1× TaqMan Universal Master Mix, and water. The thermal cycling began with an initial denaturation step of 10 min at 95 °C, followed by 40 cycles of denaturation at 92 °C for 15 s and annealing at 60 °C for 1 min. Allele detection was performed for 1 min at 60 °C on a Fast 7300 Real-Time System (Applied Biosystems, Foster City, CA, USA) to enable allelic discrimination. *CYP2C9* genotypes were assigned according to the presence of SNPs associated with the alleles of interest [18].

The genetic analysis of the ancestry of these participating individuals has also been previously analyzed [18]. African (AFR), European (EUR), and Native American (NAT) individual ancestry were estimated in 176 Dominican individuals by genotyping 90 ancestry-informative markers (AIMs) from the same panel as standardized in the previous RIBEF-CEIBA studies [17,18]. The AIMs genotyping was performed at the Spanish National Genotyping Center (CEGEN) from Santiago de Compostela, using iPLEX assays, followed by mass spectrometry analysis using the MassARRAY System (Agena Bioscience, San Diego, CA, USA) [18].

### 2.3. Determination of CYP2C9 pVNTR

A peripheral blood sample (5 mL) was collected into an EDTA tube from volunteers. DNA was extracted using the QIAamp*^®^* DNA Blood Kit (Qiagen, Hilden, Germany) and assessed for integrity and concentration via spectrophotometry with a NanoDrop*^®^* ND-1000 Spectrophotometer (Thermo Fisher Scientific, Inc., Greenville, NC, USA).

**Figure 1:**
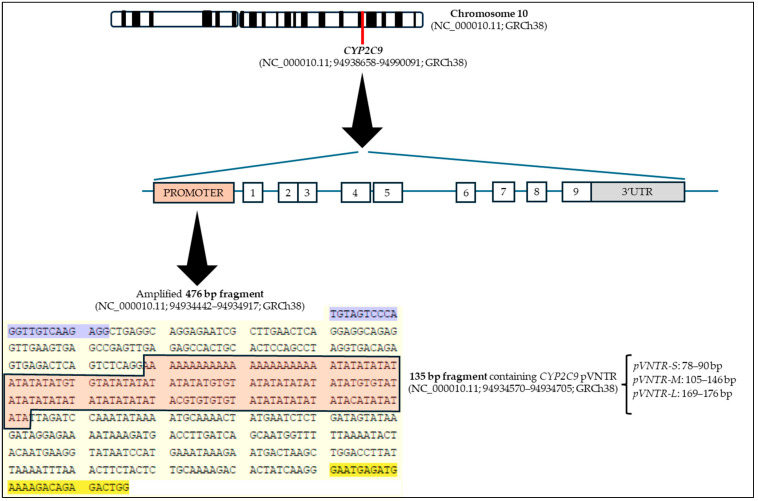
Representation and location of *CYP2C9* pVNTR. Sequence highlighted in purple: forward primer; sequence highlighted in yellow: reverse primer; pVNTR: variable number tandem repeat polymorphism; *pVNTR-S*: short; *pVNTR-M*: medium; *pVNTR-L*: long. Figure adapted from [13].

A fragment of 476 bp (NC_000010.11; 94934442–94934917; GRCh38) was PCR-amplified. This fragment contains *CYP2C9* pVNTR (NC_000010.11; 94934570–94934705; GRCh38), which is variable (Figure 1).

The sequences for the forward and reverse primers were 50-TGTAGTCCCAGGTTGTCAAGAGG-FAM-30 and 50-CCAGTCTCTGTCTTTTCATCTCATTC-30, respectively (Figure 1).

The PCRs were performed according to a previous study [15]. Briefly, initial PCR was performed using pVNTR-forward primer (10 M), pVNTR-reverse primer (10 M), and 50–80 ng of DNA in a Veriti Thermal Cycler (Thermo Fisher Scientific, Waltham, MA, USA). The PCR products were analyzed using capillary electrophoresis, and the amplification products were diluted 1:10 with Hi-Di Formamide with 0.3% (*v/v*) of GeneScan™ 600 LIZ^®^ Size Standard (Thermo Fisher Scientific, Waltham, MA, USA). Afterward the samples were denatured, and the denatured PCR products were electrophoresed using POP-7 polymer (Thermo Fisher Scientific, Bedford, MA, USA) in an Applied Biosystems Sanger Sequencing 3500 Series Genetic Analyzer (Thermo Fisher Scientific, Waltham, MA, USA), with GeneScan Analysis v5.0 (Applied Biosystems, Thermo Fisher, Waltham, MA, USA) to analyze and calculate the molecular size of the amplified alleles.

### 2.4. Data Analysis

To evaluate the relationship between ancestry and the presence of the three *CYP2C9* pVNTR variants, a Student’s *t*-test for independent samples was performed using R software (version 4.2.2; https://www.r-project.org/; accessed on 1 February 2025) with the t.test() function from the R base package, with the aim of comparing the means of the percentage of ancestry of individuals who were carriers and non-carriers of each pVNTR variant. To evaluate the normality of the variables, a Shapiro–Wilk test was performed using the shapiro.test() function.

For genotypic frequencies, the Hardy–Weinberg equilibrium was determined by comparing them with expected values using a χ^2^ contingency table statistic with Yate’s correction. To compare differences in allele frequencies of *pVNTR* polymorphisms in different populations, Fisher’s exact test was used.

Linkage disequilibrium analysis was performed using SNPstat software v1 (https://www.snpstats.net/; accessed on 14 February 2025).

*p* values less than 0.05 were regarded as statistically significant.

## 3. Results

### 3.1. Frequency of CYP2C9 pVNTR in the Dominican Population

The frequency of *CYP2C9* pVNTR was determined in a total of 193 samples (4 samples did not amplify), and the results are shown in Table 1. The presence of CYP2C9*2, *3, and *5 alleles was previously analyzed in these subjects [18], showing frequencies of 0.114, 0.034, and 0.002, respectively.

On the other hand, the analysis of pVNTR showed three different fragment sizes: 510–517 bp (*pVNTR-L*), 446–487 bp (*pVNTR-M*), and bp 419–431 bp (*pVNTR-S*), depending on the size of the 135 bp fragment containing the *CYP2C9* pVNTR (see Figure 1).

The frequencies of different fragment sizes of *CYP2C9 pVNTR-L*, *M*, and *S* found in the Dominican population were 0.065, 0.896, and 0.039, respectively (Table 1 and Table 2).

In addition, according to the frequency of *CYP2C9* pVNTR alleles in different populations, it can be observed that the frequency of the short *pVNTR* fragment in the Dominican population (0.039) is the lowest when comparing to other populations, such as the Jordanians (0.295; *p* < 0.0001) [16], Egyptians (0.115; *p* < 0.0001) [13], and Spaniards (0.081; *p* = 0.026) [15].

In contrast, the *pVNTR-M* variant presented the highest frequency of all populations studied (Table 2), whereas for the *pVNTR-L* variant, the frequency in the Dominican population was significantly lower than that found in the two White American populations previously studied (6.5% vs. 13.7% and vs. 15.2%; *p* < 0.005 and <0.0001, respectively) and in the average of those found in other populations (6.5% vs. 4.5–10.3%) (Table 2).

### 3.2. Linkage Disequilibrium Analysis Between CYP2C9 pVNTR-S and CYP2C9*3 in the Dominican Population

In the Dominican population, 83.3% (10/12) of individuals carrying the CYP2C9*3 allele also carried *pVNTR-S*. However, not all individuals carrying the *pVNTR-S* fragment also had the CYP2C9*3 allele (66.7%; 10/15), since five individuals with *pVNTR-S* were CYP2C9*1/*1 (Table 1).

In the Dominican population, both the LD coefficient (D’) between the *pVNTR-S* fragment and CYP2C9*3 and the squared correlation coefficient (r^2^) were lower (D’= 0.756, r^2^ = 0.702) than in populations with European ancestry (Table 2). Furthermore, the studied populations that also appear to present a less strong association between the two polymorphisms using the r^2^ value are those without a high EUR ancestry (African American and Egyptian; Table 2), suggesting the influence of other factors.

The analysis of LD between pVNTR and other *CYP2C9* alleles analyzed (*2, *3, *5, *6, *8) were not significant in this population, similar to that observed in other populations [13,14,15].

### 3.3. CYP2C9 pVNTR Ancestry Analysis in the Dominican Population

Linear regression analysis could not be performed in this population to analyze the association between *CYP2C9* pVNTR variants and genetic ancestry because data from other parental populations were not available to implement the analysis.

**Figure 2:**
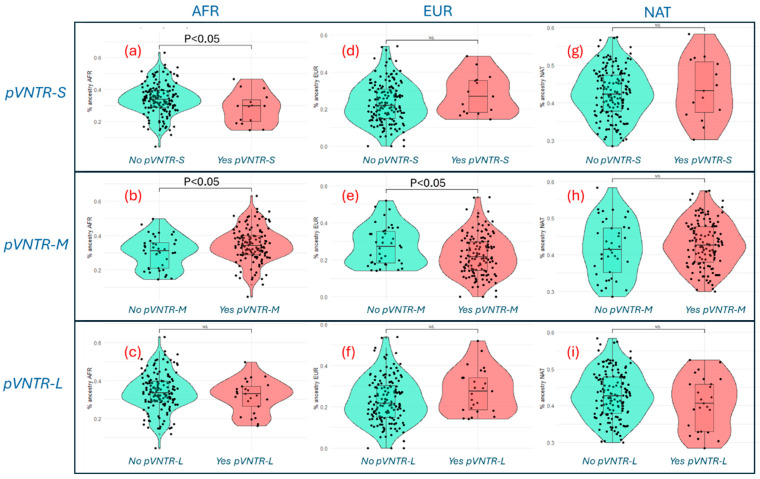
Distribution of the three *CYP2C9* pVNTR variants according to different frequencies of genetic ancestries in the Dominican population (*n* = 176). *p* < 0.05 by Student’s *t*-test. NS: not significant; AFR: African; EUR: European; NAT: Native American. (**a**) AFR *pVNTR-S*; (**b**) AFR *pVNTR-M*; (**c**) AFR *pVNTR-L*; (**d**) EUR *pVNTR-S*; (**e**) EUR *pVNTR-M*; (**f**) EUR *pVNTR-L*; (**g**) NAT *pVNTR-S*; (**h**) NAT *pVNTR-M*; (**i**) NAT *pVNTR-L*.

However, performing a Student *t*-test to compare across the percentages of ancestry shows how carriers of the *pVNTR-S* fragment have a lower percentage of AFR ancestry than those who are not carriers (*p* = 0.031; Figure 2a), whereas carriers of the *pVNTR-M* allele have a higher frequency of AFR ancestry than non-carriers (*p* = 0.019; Figure 2b). Conversely, *pVNTR-M* carriers have a lower percentage of EUR ancestry than non-carriers (*p* = 0.008; Figure 2e). Finally, the *pVNTR-L* allele was not significantly associated with any ancestry group (Figure 2g–i).

## 4. Discussion

This is the first study analyzing *CYP2C9* pVNTRs in a Latin American population, specifically, in the Caribbean Dominican Republic. The ancestral molecular component of this population, which has been previously studied [18], indicates that the percentage of genomic ancestry for these individuals is 23.8% EUR, 42.6% NAT, and 33.6% AFR, demonstrating that it is a highly mixed population with more than 75% non-European ancestry.

Previous studies have been conducted to analyze *CYP2C9* pVNTR in different populations, such as Egyptian, African [13], White American [13,14], Jordanian [16], and Spanish populations [15]. Regarding the frequencies of pVNTRs, the frequency of the *pVNTR-M* variant in the Dominican population was the highest among all the populations reported to date (89.6%), being similar only to that of the African American population (Table 2). As for the frequency of the long *pVNTR* fragment in the Dominican population, it was only different from that found in (Table 2) the White American populations, which can be explained in relation to the low percentage of European ancestry in the studied Dominican population (Table 2). Lastly, it is highlighted that the frequency of the short *pVNTR* fragment in the Dominican population was the lowest reported to date (3.9%; Table 2).

Regarding the LD between *pVNTR-S* and CYP2C9*3 variants, the Dominican population shows a weaker association (D’ = 0.70; r^2^ = 0.67) than in previously studied populations with European ancestry, but higher than in other populations, such as the Egyptians [13] (Table 2). Since strong LD is expected in parental populations due to a lower recombination frequency in these populations, a weaker LD was expected in Dominicans.

On the other hand, it would be more accurate the term ancestral haplotype than linkage disequilibrium, since these two variants (CYP2C9*3 and *pVNTR-S*) are found in the same gene (locus), and to establish the haplotype of each individual, further studies would be necessary to sequence the entire gene, or at least the promoter region and exon 7, which is where the pVNTR and reference SNP for the CYP2C9*3 variant are located, respectively.

Although it was not possible to perform a linear regression analysis to analyze the association between *CYP2C9* pVNTR variants and genetic ancestry, because more populations studied are needed to implement such an analysis, the present original exploratory findings support the hypothesis that carriers of the *pVNTR-S* fragment appear to have a lower percentage of AFR ancestry than non-carriers (*p* = 0.031; Figure 2a). However, this hypothesis needs to be tested by comparing the genetic ancestry of other populations.

The clinical significance of *CYP2C9 pVNTR-S* is based on findings reported in two previous studies [13,14]. In the first of them [13], it was reported that in European Americans on treatment with standard dose of warfarin, *pVNTR-S* predicted a reduction in warfarin, but the in vivo effects of *pVNTR-S* on CYP2C9 metabolism could not be separated from the effects of CYP2C9*3 due to the high LD in European populations [13]. On the other hand, the other recently published study [14] has shown an association between the presence of *pVNTR-S* and the decrease in CYP2C9 expression, independently of the effect of CYP2C9*3. That is, *pVNTR-S* was shown to have an independent effect on CYP2C9 expression, and then, it may further contribute to the reduction of CYP2C9 activity when coexisting/co-expressed with CYP2C9*3 [14].

Nevertheless, *pVNTR-S* cannot be considered an independent biomarker for CYP2C9 activity in populations with a strong coefficient of LD (high D’ and r^2^) with CYP2C9*3; however, in cases where *pVNTR-S* does not coexist with CYP2C9*3, such as in individuals of non-European descent, *pVNTR-S* may serve as an additional biomarker to predict the reduction of CYP2C9 activity. Thus, according to this proposal, *pVNTR-S* may improve *CYP2C9* genetic testing panels for personalized drug therapy in populations [19] where the LD between *pVNTR-S* and CYP2C9*3 is not too high, such as in the Dominican population, which requires the performance of validation studies in humans.

## 5. Conclusions

This is the first study that analyzes the frequency and presence of *CYP2C9* pVNTR in a Latin American population, specifically, in a Caribbean population from the Dominican Republic. Results show a lower *CYP2C9 pVNTR-S* frequency than the ones reported in the rest of the populations published in studies to date, and, also, analysis of the presence of *CYP2C9 pVNTR-S* and CYP2C9*3, such as an ancestral haplotype, might be of help in understanding and predicting the reduction of CYP2C9 activity in populations with different ancestries.

Further research into *CYP2C9* pVNTR is needed, including studies with larger and more diverse populations around the world. In addition, clinical studies, both phenotyping and measuring drug concentrations, would be necessary to test the independent effect of *CYP2C9* pVNTR in humans, mainly in individuals without known variants with decreased CYP2C9 activity (such as *3, *5, *6*, *8, etc.), but carriers of the *pVNTR-S* fragment.

## Figures and Tables

**Table 1:** Percentage of *CYP2C9* star allele genotypes and pVNTR in the Dominican population (*n* = 193).

			*CYP2C9* pVNTR					
			*L*/*M*		*M*/*M*		*M*/*S*	
*CYP2C9*	*n*	%	*n*	%	*n*	%	*n*	%
*1/*1	141	73.1	21	10.9	115	59.6	5	2.6
*1/*3	9	4.6	-	-	2	1.0	7	3.6
*1/*5	1	0.5	-	-	1	0.5	-	-
*1/*2	36	18.7	4	2.1	32	16.6	-	-
*2/*2	3	1.6	-	-	3	1.6	-	-
*2/*3	2	1.0	-	-	-	-	2	1.0
*3/*3	1	0.5	-	-	-	-	1	0.5
			25	13.0	153	79.3	15	7.7

*L*: long; *M*: medium; *S*: short.

**Table 2:** Allele frequencies of *CYP2C9* pVNTR and linkage disequilibrium (LD) analysis between pVNTR-S and CYP2C9*3 alleles in different populations.

Population	*n*	CYP2C9*3	*pVNTR-S*	*pVNTR-M*	*pVNTR-L*	^#^D’ (r^2^)	Ref.
African American	134	0.010	0.213 ***	0.742 ***	0.045	0.99 (0.05)	[14]
African American	120	0.025	0.051	0.883	0.065	n.p. (0.53)	[13]
Egyptian	207	0.092	0.115 ***	0.785 ***	0.100	n.p. (0.59)	[13]
Jordanian	205	n.e.	0.295 ***	0.627 ***	0.078	n.e.	[16]
Spanish	209	0.074	0.081 *	0.816 **	0.103	0.93 (0.88)	[15]
White American	804	0.050	0.058	0.789 ***	0.152 ***	n.p. (0.75)	[13]
White American	113	0.040	0.049	0.814 **	0.137 **	0.99 (0.79)	[14]
Dominican	193	0.034	0.039	0.896	0.065	0.76 (0.70)	Present study

*** *p* value < 0.0001; ** < 0.005; * < 0.05 by two-tailed Fisher exact test compared to Dominicans; in bold type statistically significant; ^#^D’ (r^2^) refers to *pVNTR-S* and CYP2C9*3; n.e. = not evaluated; n.p. = not provided.

## Data Availability

The original contributions presented in this study are included in the article. Further inquiries can be directed to the corresponding author.
